# Influence of Teaching Style on Physical Education Adolescents’ Motivation and Health-Related Lifestyle

**DOI:** 10.3390/nu11112594

**Published:** 2019-10-29

**Authors:** Rubén Trigueros, Luis A. Mínguez, Jerónimo J. González-Bernal, Maha Jahouh, Raul Soto-Camara, José M. Aguilar-Parra

**Affiliations:** 1 Department of Psychology, Hum-878 Research Team, Health Research Centre, University of Almeria, 04120 Almeria, Spain; 2 Department of Psychology, University of Burgos, 09001 Burgos, Spain; laminguez@ubu.es (L.A.M.); jejavier@ubu.es (J.J.G.-B.); mjx0002@alu.ubu.es (M.J.); rscamara@ubu.es (R.S.-C.)

**Keywords:** Physical Education, teacher, motivation, healthy habits, structural dimensions

## Abstract

According to various WHO reports in 2018, a large number of adolescents worldwide are either overweight or obese. This situation is the result of not following a healthy and balanced diet, combined with a lack of practice of physical activity. In this sense, Physical Education classes could help to solve the problem. The present study seeks to analyze the relationship between the role of the teacher in relation to the structural dimensions of the PE teaching environment and the basic psychological needs and self-motivation of adolescents as determinants of their behaviors related to eating habits and the practice of physical activity. A total of 1127 secondary school adolescents between the ages of 13 and 18 participated in this study. Questionnaires were used: Perceived Autonomy Support Scale, Psychologically Controlling Teaching Scale, Basic Psychological Needs in Physical Education, Frustration of Psychological Needs in PE context, Physical Activity Class Satisfaction Questionnaire, Perceived Locus of Causality Revised, and WHO’s Global school-based student health survey. A structural equations model was elaborated to explain the causal relationships between the variables. The results showed that autonomy support positively predicted the three structural dimensions of PE classes, while, in contrast, they were negatively predicted by psychological control. The three structural dimensions positively predicted the satisfaction of psychological needs and negatively predicted the thwarting of psychological needs. Self-determined motivation was positively predicted by the satisfaction of psychological needs and negatively predicted by the thwarting of psychological needs. Finally, self-determined motivation positively predicted healthy eating habits and the practice of physical activity and negatively predicted unhealthy eating habits. Certainly, the results obtained in this study support the postulates of the self-determination theory, demonstrating the predictability of PE class context towards the adoption of healthy lifestyle habits, such as a proper diet and the regular practice of physical activity.

## 1. Introduction

According to the WHO report [1], more than 276 million adolescents (12 to 19 years old) are overweight or suffer from obesity worldwide. This situation can be linked to the fact that our diets include more and more high-calorie foods that are also high in fat, compounded with a decrease in physical activity (PA) due to a more sedentary lifestyle. These data are even more alarming if we consider another WHO report [2], which reached the conclusion that 80% of the adolescent population does not practice in any type of physical sports activity on a regular basis. Indeed, a sedentary lifestyle combined with an unhealthy diet constitutes an important risk factor for the development of chronic diseases, obesity, depression, and anxiety, causing problems at psychological, physical, and emotional levels [3]. Therefore, PE classes represent the ideal medium for the education and overall training of adolescents, which helps them to consolidate active lifestyle habits that will last throughout the rest of their lives. In this regard, according to Spanish Education Law (LOMCE; Ley Organica de la Mejora de la Calidad Educativa), one of the basic objectives of PE classes is to consolidate healthy lifestyle habits, in terms of both diet and responsible PA [4]. Thus, the present study intends to analyze how PE classes can help to consolidate such habits during adolescence.

According to the Self-Determination Theory (SDT; [5]), individuals can be influenced by the social context that surrounds them via two completely different interpersonal styles: controlling style versus autonomy support. The former refers to the use of external pressures, impositions, threats, or punishments, among others, which are seen as the origin of the behaviors manifested by adolescents, negatively affecting personal response, self-knowledge, and effort [6]. In contrast, autonomy support deals with self-initiative, personal self-adjustment, and the mental and physical development of the adolescent [7]. However, SDT not only focuses on interpersonal styles but also on the structure of the environment, which ultimately determines how social agents provide clear, contingent, and coherent directions in the learning context [8]. With regard to PE, structure would involve the class lesson plan, class organization, how activities develop in class, the clarity and quality of teacher feedback, and the practical use of different abilities, among others. Therefore, if a teaching environment is well structured, it is more likely that adolescents will perceive they are learning concepts, improving their motor skills and health, and creating new bonds with classmates [9,10]. For this reason, during classes, it is necessary for the teacher to: (A) provide adolescents with a positive mastery experience, which implies the development and improvement of physical abilities; (B) foster their cognitive development, referring to the development and improvement of cognitive lessons; and (C) successfully execute teaching, which refers to the level of satisfaction with the teaching by the adolescents within the class content [11].

According to SDT, social context influences psychological needs that are defined as essential elements for well-being and personal development [12]. More specifically, there are three psychological needs: autonomy, competence, and relatedness [13]. Autonomy has been defined as the desire to feel that one is the origin and regulator of their own behavior. Competence is the individual’s perception that they are capable of demonstrating effectiveness within a particular context. Finally, relatedness refers to the feeling one has of belonging to a specific social environment. However, very recent studies have incorporated novelty as another psychological need, defined as the individual’s pursuit of new and different experiences and activities that promote personal development and well-being [14,15]. 

Essentially, adolescents feel autonomous when they make their own decisions, competent when their abilities meet the challenge, integrated into a given social group, and stimulated when activities are different and appealing. In these circumstances, adolescents will feel their psychological needs are satisfied, thereby allowing them to experience self-determined motivation, which is associated with acquiring new motor skills and contents, learning, improving relationships with classmates, and tuning and acquiring positive adaptive behaviors [5]. On the other hand, if in PE classes adolescents perceive a sense of neglect, excessively difficult or easy challenges, lack of control over their decisions, and tedious activities, adolescents will experience frustration of their psychological needs. As a result, they will tend to perceive non-autonomous motivation or even demotivation, which is associated with a lack of commitment, quitting an activity, a decrease in interpersonal relationships with classmates, and the adoption of negative behaviors [16]. 

Both social context and psychological needs can significantly influence the motivation of the adolescent towards PE classes [5,7]. According to SDT, motivation can be either non-autonomous or autonomous. The former is related to participation in activities due to acquired obligations or external pressures. Conversely, self-determined motivation is linked to behaviors based on personal initiative and choice. This second type of motivation facilitates the individual’s adaptation because it leads to self-regulation of behavior as people tend to persevere due to the personal satisfaction that the activity produces. In contrast, non-autonomous motivation fosters maladaptive behavior as individuals tend to avoid an activity if they are not rewarded [13]. 

To date, existing studies in the field of PE classes have mainly focused on the clear perspective or, in other terms, the positive vertex of SDT. Such works have analyzed the influence of autonomy support on the satisfaction of psychological needs [17], self-motivation towards PE classes [18], and the influence of the latter on the adoption of healthy lifestyle habits [19]. Thus, in recent years, important lines of research have emerged that concentrate on the dark perspective of SDT or, in other words, the negative vertex [20]. This dark vertex has revealed that the controlling teaching style has negative effects on the satisfaction of psychological needs [21], self-determined motivation [22], resilience [23], and learning [24]; and positive effects on the frustration of psychological needs [21]. However, studies focusing on the dark perspective of SDT are rather scarce, and even more so are those investigations that analyze both types of teaching approaches (autonomy support versus controlling style). As for other research, studies which have analyzed the influence of social context have mainly focused on adolescents’ perception of their teacher and not on their perceptions of the structured PE learning environment, when in fact the latter proves fundamental to gaining greater understanding of the relationships between behaviors and results achieved (e.g., healthy eating habits and regular practice of physical activity) by adolescents.

In this sense, it is important to highlight the importance of PE classes towards promoting a healthy and balanced diet and an active lifestyle through a learning climate and the participation of teachers [25]. In this sense, the PE curricula in different EU countries establish that adolescents should receive knowledge related to healthy living habits (diet and PA practice) and motor skills in the aim of improving their health and quality of life [26]. In this way, different studies in the context of Physical Education have shown their relationship with the adoption of active habits by adolescents outside the school context [27]. Similarly, different studies suggest that PE classes have a significant influence on the adoption of a healthy and balanced diet [28].

**Figure 1:**
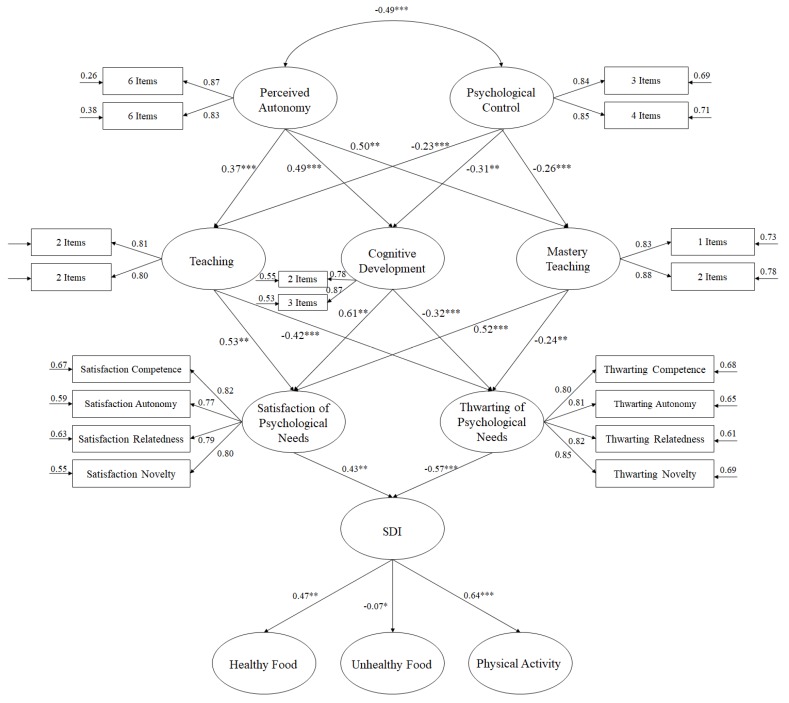
Hypothesized model, where all variables are related to one another. All parameters are statistically significant and are standardized. Note: * *p* < 0.05; ** *p* < 0.01; *** *p* < 0.001.

Taking into account the postulates of SDT and the previously-mentioned points, the present study aims to analyze the relationship between the role of the teacher in relation to the dimensions of the structured environment of PE teaching and the basic psychological needs and self-motivation of adolescents as determining factors of behaviors related to eating habits and the practice of physical activity. Thus, we propose the following hypotheses (see Figure 1): (1) autonomy-support by the teacher will positively predict teaching, cognitive development, and teaching experiences; (2) the controlling teaching style will negatively predict teaching, cognitive development, and teaching experience; (3) teaching, cognitive development, and mastery experiences will positively predict the satisfaction of psychological needs and negatively predict the thwarting of psychological needs; (4) the satisfaction of psychological needs will positively predict autonomous motivation; (5) the thwarting of psychological needs will negatively predict autonomous motivation; (6) autonomous motivation will positively predict healthy eating habits and participation in physical activity and negatively predict unhealthy eating habits. 

## 2. Method

### 2.1. Participants

A total of 1127 secondary school adolescents participated in this study, of whom 653 were male and 474 were female. The participants were between the ages of 13 and 18 (M = 15.27; SD = 1.35), and came from various secondary schools from the Spanish provinces of Burgos (45.78%) and Almeria (54.22%). The classes were carried out respecting the equal rights and duties of the adolescents. Participation in the study was voluntary but, in order to do so, written authorization from parents or legal guardians was required. Furthermore, all adolescents completed the questionnaires, as one of the criteria for inclusion in the study was to fill in each of the scales.

The sampling used was incidental non-probabilistic, based on those educational centers and adolescents to which access was obtained.

### 2.2. Instruments

#### 2.2.1. Perceived Autonomy Support

The Spanish version of the Perceived Autonomy Support Scale for Exercise Settings by Hagger et al., [29], which was adapted and validated for the context of PE in Spain by Moreno, Parra, and González-Cutre [24]. There are 12 items that compose this scale, which evaluate only one factor of autonomy support (i.e., I am made to feel guilty when I disappoint my teacher). This tool scores responses using a Likert scale from totally disagree (1) to totally agree (7). 

#### 2.2.2. Psychological Control

This aspect was measured using the Psychologically Controlling Teaching Scale (PCTs; Soenens, et al., [30]), validated and adapted by Trigueros, Aguilar-Parra, González-Santos and Cangas [6] to the context of Physical Education. The scale featured the heading “My Physical Education teacher…”. The questionnaire consisted of 7 items with one single factor (i.e., I am made to feel guilty when I disappoint my teacher). Adolescents had to respond according to a Likert scale, which ranged from totally disagree (1) to totally agree (5).

#### 2.2.3. Satisfaction of Basic Psychological Needs

The tool used in this case was the version of Basic Psychological Needs in Physical Education (BPN-PE; [31]), adapted and validated to the Spanish PE context by Menéndez and Fernández-Río [32], to which Trigueros, Aguilar-Parra, Cangas, Álvarez, and González-Santos [33] integrated the items corresponding to novelty developed by González-Cutre et al., [15]. The scale is comprised of a total of 18 items divided among four factors: 4 correspond to autonomy, 4 correspond to competence, 4 items correspond to relatedness to others, and 6 items correspond to novelty. Adolescents responded according to a Likert scale from totally disagree (1) to totally agree (7). 

#### 2.2.4. Thwarting of Psychological Needs

In this case, the present adolescent utilized the version of the Scale for the Frustration of Psychological Needs in physical exercise by Sicilia, Ferriz and Sáenz-Álvarez [34], validated by and adapted to the Spanish PE context by Trigueros, Maldonado, Vicente, González-Bernal, and González-Santos [14]. The scale features the heading “In my PE classes…” and consists of 17 items, which are divided among the scale factors as follows: 4 items for autonomy, 4 items for competence, 4 items for relatedness to others, and 5 items for novelty. Adolescents responded according to a Likert scale ranging from not true at all (1) to totally true (7). 

#### 2.2.5. Perceived Structured PE Teaching Environment

This aspect was measured using the version of the Physical Activity Class Satisfaction Questionnaire [10], adapted and validated to the Spanish PE context by Sicilia, Ferriz, Trigueros and González-Cutre [11]. The questionnaire consists of 45 items divided among 9 factors and features the heading: “Indicate your level of satisfaction with Physical Education classes you’ve received regarding…”. However, to measure the satisfaction of adolescents with regard to knowledge of theory, acquired skills, and teaching style, only three factors were utilized: cognitive development, mastery experiences, and teaching. Adolescents indicated their responses according to a Likert scale from totally disagree (1) to totally agree (8). 

#### 2.2.6. Motivation

The instrument utilized was the version of the Perceived Locus of Causality Revised (PLOC-R) by Vlachopoulos et al. [35], adapted and validated to the Spanish PE context by Trigueros, Sicilia, Alcaraz and Dumitru [4]. The scale featured the heading “I participate in Physical Education class…” and is comprised of 23 items grouped among six factors, which measure amotivation, external regulation, introjected regulation, identified regulation, integrated regulation, and intrinsic motivation. The pupils responded according to Likert scale that ranged between not true at all (1) and totally true (7).

Autonomous motivation was also evaluated using the self-determination index (SDI; [36]). The latter was calculated based on the following formula: 3 × intrinsic motivation, 2 × integrated regulation, 1 × identified regulation, −1 × introjected regulation, −2 × external regulation, and −3 × amotivation. Various works have shown this index to be valid and reliable, and it is applied to obtain a value that facilitates quantifying that level of self-determination.thy and Unhealthy Eating Habits and Participation in Physical Activity

The tool utilized was the Spanish version [37] of the WHO’s Global school-based student health survey [38]. For the purposes of this study, we selected indices related to healthy foods (such as fruit, fish, and vegetables) and unhealthy foods (such as candy, snacks, and pastries) consumed on a weekly basis. An index was calculated, which ranged from hardly ever (1) to every day (4). As for participation in physical activity, an index was calculated according to the number of days per week for each physical activity and the duration of each session. This index ranged from 1 to 6. For a more detailed explanation of these indices and their validity, see Balaguer [37]. 

### 2.3. Procedure

Firstly, the schools are contacted to ask for their collaboration. Later, the adolescents were asked for informed consent signed by their parents or legal guardians so that they could participate in the study. The PE teachers were then informed of the purpose of the study and that the questionnaires would be administered before the start of classes. Finally, the adolescents were informed that they would participate in research on motivation and healthy habits. This study was conducted according to the recommendations of the American Psychology Association. The experiment was carried out in compliance with the Declaration of Helsinki. Ethics approval was obtained (Ref. UALBIO 2019/014) from the Bioethics Committee for Human Research of the University of Almería.

### 2.4. Data Analysis

Analyses were performed on the descriptive statistics and the bivariate correlations, in addition to a reliability analysis, using the statistics program SPSS 25. Furthermore, Structural Equations Modelling (SEM) was constructed with the statistics program AMOS 20.

In order to develop the hypothesized model (Figure 1), a maximum likelihood estimation was utilized, along with a bootstrapping procedure. The estimators were not affected by the lack of normality, meaning they were considered robust. With the aim of assessing the tested model, various fit indices were considered: χ^2^/df, Incremental Fit Index (IFI), Comparative Fit Index (CFI), Standardized Root Mean Square Residual (SRMR), and Root Mean Square Error of Approximation (RMSEA) plus its confidence interval (CI) at 90%. Values less than 3 for χ^2^/df, values for CFI and IFI greater than or close to 0.95, and values for SRMR and RMSEA very close or less than to 0.06 and 0.08 were considered, respectively, as indicating a suitable fit of the model to the data [39]. 

However, these adjustment indices should be interpreted with caution, as they are too difficult and restrictive when analyzing complex models [40].

## 3. Results

### 3.1. Preliminary Analysis

Table 1 displays the bivariate correlations, the average and typical deviation and the reliability analysis conducted using Cronbach’s alpha for each of the study variables: autonomy support, psychological control, teaching, cognitive development, mastery experiences, satisfaction of psychological needs, frustration of psychological needs, self-determination index, healthy eating habits, unhealthy eating habits, and physical activity. 

As for the correlation analyses, it can be observed that there was a positive correlation between autonomy support, teaching, cognitive development, teaching experiences, satisfaction of psychological needs, SDI, healthy eating habits, and physical activity. These analyses also revealed negative correlations between psychological control, frustration of psychological needs, and unhealthy eating habits. In addition, there was a positive correlation between psychological control, frustration of psychological needs, and unhealthy eating habits, while there was a negative correlation with autonomy support, teaching, cognitive development, teaching experiences, satisfaction of psychological needs, self-determination index, healthy eating habits, and physical activity.

### 3.2. Structural Equations Modelling Analysis

Due to the complexity of the model, the number of indicators was reduced by at least two to analyze the relationships between the model variables. Subsequently, through an SEM, the hypothesized model was tested [41]. More specifically, the existing variables used were: frustration of basic psychological needs, which included four indicators (frustration of competence, autonomy, relatedness to others, and novelty) [14]; satisfaction of basic psychological needs, which included four factors (satisfaction of competence, autonomy, relatedness to others, and novelty) [33]; and, finally, in the case of autonomy support, it was necessary to divide the 12 items on the scale into two indicators, as were the cases with the 7 items pertaining to psychological control, the 5 items of cognitive development, the four items of teaching, and the 3 items of teaching experiences. This procedure was followed to be able to identify the model [41].

The model for the hypothesized predictive relationships (Figure 1) revealed the following fit indices: χ^2^ (215, N = 1127) = 641.35, CFI = 0.95, IFI = 0.95, χ^2^/df = 2.98, *p* < 0.001, RMSEA = 0.053. (IC 90% = 0.049 − 057), SRMR = 0.046. The hypothesized model can be considered appropriate since the results are in compliance with the established parameters. In addition, the influence of each factor with respect to other variables was analyzed using standardized regression weights. 

## 4. Discussion

The purpose of the present study was to analyze how the interpersonal style of the teacher influences the dimensions of the structured PE teaching environment and the basic psychological needs and self-motivation of adolescents as determinants of their behaviors related to eating habits and the practice of PA. This study is the first to consider the role of the teacher, in terms of the duality of autonomy support versus controlling style, in the three structural dimensions of PE classes and, in turn, the influence of the latter on psychological needs (satisfaction and frustration). This dual role of the teacher and of the structured teaching environment proves essential given the influence the teacher commands over the emotional, social, and psychological development of adolescents [7]. In addition, this influence is critical in determining how adolescents develop their abilities and knowledge based on a balance between discovery and past experiences, which allow them to make better decisions in their lives, both in the present and future [42]. Moreover, the present work analyzes the influence of PE classes on how adolescents make decisions related to specific aspects of their lifestyles, more specifically eating habits and the regular practice of physical activity. In this regard, the subject of PE is an ideal discipline for ensuring that adolescents develop knowledge and a set of abilities and attitudes which favor the adoption of healthy lifestyle habits.

The results demonstrate that autonomy support positively predicted the three structural dimensions of PE classes (teaching, cognitive development, and mastery experience). Conversely, psychological control negatively predicted the three structural dimensions. Results of studies similar to the present study conducted during the secondary school phase also showed that autonomy support is positively related to PE class structure [43,44]. However, there are hardly any examples of previous studies in the field of PE that link the controlling teaching style to the three structural dimensions of classes. In this sense, the present study sought to address certain limitations of past investigations that analyzed the influence of social context in the teaching field, more specifically, PE. In our case, a model was presented in which not only both roles of the teacher are included but also the structural dimensions and the existing relationships between the teacher’s role and the structure. Thus, the results between the dual role of the teacher and PE class structures suggest that when teachers provide directions, rules, and comments necessary for guiding adolescent behavior, teachers tend to employ an autonomy support style. Furthermore, teachers who support autonomy try to respect their adolescents’ internal frame of reference, and highly structured teachers try to promote feedback, assistance, knowledge about concepts and motor skills based on suitable challenges, which could explain why teachers who are perceived as partisan to autonomy support are also more prone to be highly structured [45]. The opposite would occur with psychological control by the teacher, given that when teachers tend to utilize unrealistic challenges and negative comments and feedback in their classes, they are not viewed by adolescents as structured. 

The results also demonstrate that the three structural dimensions of PE classes positively predict the satisfaction of psychological needs and negatively predict the frustration of psychological needs. These findings are similar to those of previous studies in the field of PE, in particular, a study carried out by Ferriz, et al. [46] with PE adolescents in secondary school, which found that structure was positively related to the satisfaction of psychological needs. Nonetheless, hardly any research can be found that has related structure to the frustration of psychological needs. Thus, the present study aimed to address specific limitations of past investigations. In this sense, the frustration of psychological needs can generate a series of maladaptive behaviors among PE adolescents [47], which is why it is crucial to understand how these needs are generated in order to prevent them. The results of the present study on the relationship between PE class structures and the satisfaction and frustration of psychological needs reveal that those teachers who stimulate the learning of concepts, technical aspects, and fundamentals of PE and who increase adolescents’ motor skills through integration and innovative methods make adolescents feel more autonomous, competent, and interested in their PE classes [44]. In this regard, adolescents with more knowledge and motor skills, and who have been exposed to enriching lessons, will perceive themselves as competent enough to carry out PE activities effectively. Possessing this attitude will motivate them to make their own decisions, both individually and in groups, and involve themselves more actively in the teaching-learning process, thereby fostering a greater perception of autonomy and connection to others [48]. In addition, the learning of new physical abilities and the utilization of innovative methods in teaching can be linked to the satisfaction of psychological needs, given that the various motor skills proposed during PE classes are practiced in groups and seek cooperative learning, thus increasing the satisfaction of competence, relatedness to others, and novelty [49]. 

The results also showed that the thwarting of psychological needs negatively predicted autonomous motivation, whereas the satisfaction of psychological needs positively predicted autonomous motivation. These results are similar to multiple previous studies [42,50,51], where feeling confident and competent during class exercises, feeling integrated and a good interpersonal relationships with classmates and/or with the teacher, feeling ownership of one’s own destiny, and participating in original activities helps adolescents to feel an autonomous motivation towards PE classes [52]. Following this, autonomous motivation positively predicted healthy eating habits and the practice of physical activity and negatively predicted unhealthy eating habits. As for other results, autonomous motivation positively predicted healthy eating habits and participation in physical activity and negatively predicted unhealthy eating habits. These findings are similar to the studies conducted by Digelidis, Papaioannou, Laparidis, and Christodoulidis [53] and Hagger and Chatzisarantis [54] who established that motivation towards PE classes positively predicts the practice of physical activity with secondary school adolescents.

On the other hand, no studies were found that analyzed the relationship between autonomous motivation and unhealthy eating habits, yet there was a study that examined the relationship existing between the PE context and both healthy and unhealthy eating habits. However, a study conducted by Jiménez-Castuera, Cervelló, García-Calvo, Santos-Rosa, and Iglesias-Gallego [55] with adolescents showed that those who had a high level of involvement in Physical Education classes showed a greater predisposition towards the practice of physical activity outside the school context and the adoption of a healthy and balanced diet. Therefore, the results of this work suggest that if PE classes stimulate knowledge of theory and physical learning through methods that integrate, adolescents will be positively encouraged to engage in physical activity, maintain healthy eating habits, and reject unhealthy ones.

Finally, it should be noted that Pearson’s analysis showed a strong correlation between PA and eating healthy food. This result is similar to multiple studies. In this sense, a study carried out by Pyper, Harrington, and Manson [56] showed that those adolescents who practice physical activity showed a greater predisposition towards a healthy and balanced diet and vice versa. These results can be explained by the fact that those adolescents who are engaged in regular physical activity in order to have a better quality of life showed a greater predisposition towards the adoption of a healthy and balanced diet based on the consumption of healthy foods that contribute to an improvement in physical and personal well-being.

As for the limitations of the present study, it should be noted that it is a correlational study whose results can be interpreted in a different way according to the reader’s understanding so that cause-effect relations cannot be established. Therefore, in order to explain the relationships between the variables of the study, we have tried to present possibilities. Therefore, future research should analyze in detail the relationships established between the variables of the study, for example, through intervention studies. In addition, it would also be interesting to determine the influence of the teacher’s pro-social skills on the structural dimensions and, in turn, their influence on adolescents’ resilience and motivation, for the purpose of ascertaining their effect on the adoption of healthy adaptive behaviors. 

## 5. Conclusions

The results obtained in the present study are in line with the postulates of SDT, demonstrating the importance and predictability of PE class context towards the adoption of healthy lifestyle habits, such as diet and the regular practice of physical activity.

## Figures and Tables

**Table 1:** Correlations between all variables and descriptive statistics.

Factors	*M*	*SD*	α	1	2	3	4	5	6	7	8	9	10	11
1. Autonomy Support	4.11	0.68	0.89	-	−0.51 ***	0.62 ***	0.44 ***	0.37 ***	0.48 ***	−0.21 **	0.34 **	0.10 *	−0.14 *	0.23 **
2. Psychological Control	1.36	1.00	0.83		-	−0.26 **	−0.23 **	−0.31 **	−0.52 **	0.60 ***	−0.48 ***	−0.28 ***	0.12 **	−0.36 ***
3. Teaching	5.82	1.08	0.78			-	0.59 ***	0.70 ***	0.47 **	−0.22 ***	0.63 ***	0.34 **	−0.11 *	0.24 **
4. Cognitive Development	4.67	1.43	0.82				-	0.20 ***	0.45 ***	−0.31 **	0.64 ***	0.27 **	−0.23 **	0.42 ***
5. Mastery Experiences	5.23	1.97	0.80					-	0.28 **	−0.39 ***	0.54 **	0.29 *	−0.18 **	0.37 **
6. Satisfaction PN	5.32	0.81	0.87						-	−0.56 ***	0.75 ***	0.38 **	−0.13 *	0.49 ***
7. Thwarting PN	1.76	1.47	0.85							-	−0.37 ***	−0.19 **	0.27 ***	−0.38 **
8. SDI	12.95	13.62	-								-	0.51 ***	−0.14 **	0.67 ***
9. Healthy Food	2.79	0.77	-									-	−0.16 ***	0.69 ***
10. Unhealthy Food	1.92	0.87	-										-	−0.08 *
11. Prac. Physical Activity	3.79	1.24	-											-

Note: PN = Psychological Needs; SDI = Self-Determination Index; Prac. = Practice; *** *p*< 0.001; ** *p* < 0.01; * *p* < 0.05.
